# Subspace-based self-interference cancellation for full-duplex MIMO transceivers

**DOI:** 10.1186/s13638-017-0839-x

**Published:** 2017-03-23

**Authors:** Ahmed Masmoudi, Tho Le-Ngoc

**Affiliations:** Department of Electrical and Computer Engineering, McGill University, Montreal, Quebec Canada

**Keywords:** Full-duplex communication, SI suppression, MIMO, parameter estimation, Subspace method, Second-order statistics

## Abstract

This paper addresses the self-interference (SI) cancellation at baseband for full-duplex MIMO communication systems in consideration of practical transmitter imperfections. In particular, we develop a subspace-based algorithm to jointly estimate the SI and intended channels and the nonlinear distortions. By exploiting the covariance and pseudo-covariance of the received signal, we can increase the dimension of the received signal subspace while keeping the dimension of the signal subspace constant, and hence, the proposed algorithm can be applied to most of full-duplex MIMO configurations with arbitrary numbers of transmit and receive antennas. The channel coefficients are estimated, up to an ambiguity term, without any knowledge of the intended signal. A joint detection and ambiguity identification scheme is proposed. Simulation results show that the proposed algorithm can properly estimate the channel with only one pilot symbol and offers superior SI cancellation performance.

## Introduction

Half-duplex transmission is commonly used in the current communication systems by transmitting and receiving over orthogonal channels. Full-duplex communication represents an attractive alternative to save channel resources or to increase the transmission efficiency. The main deterrent to employ full-duplex is the large self-interference (SI) from the simultaneous transmission and reception over the same frequency band. The SI is usually several orders of magnitude higher than the intended signal received from the other transmitter, because the later travels a longer distance than the former signal. Recent works have shown that, using different cancellation stages, the SI can be sufficiently suppressed to properly detect the intended signal [1, 2].

The SI is first cancelled at the radio-frequency (RF) level, prior to the low-noise amplifier (LNA) and the analog-to-digital converter (ADC), to avoid overloading/saturation of these devices [1–3]. In other words, the SI should be sufficiently suppressed at RF to maintain the receiver’s limited dynamic range. Then, further SI suppression can be done after the ADC at the baseband [4, 5]. In the following, we assume that a cancellation stage at RF is available and we concentrate on the SI cancellation in the baseband.

To further reduce the SI, channel state information of the interference link should be available. Therefore, estimating the SI channel is a critical issue in full-duplex systems. In [6], the SI channel estimation is performed in the frequency domain using a least square (LS) technique. LS and minimum mean square error (MMSE) channel estimations are proposed in [7] to estimate the SI channel in the relay station. However, these approaches ignore the intended signal coming from the other transceiver and treat it as additive noise. An adaptive least mean square algorithm to estimate the SI channel is proposed in [8] where the large SI compared to the intended signal and additive noise is exploited to obtain an estimate of the SI channel. A more elaborate LS-based estimator was presented in [9] where a first estimate of the SI channel is obtained by considering the intended signal as additive noise. Then an iterative detection of the intended signal and channel estimation is performed to obtain a better estimate of the channel. On the other hand, spatial domain cancellation attempts to reduce the SI by precoding at the transmit chain and decoding at the receive chain. Spatial domain cancellation is formulated in the frequency domain [10–12]. An alternative time domain formulation was presented in [13] by precoding the transmitted SI to coincide with the null space of the SI channel. These techniques are based on the knowledge of both the SI and intended channels at the two transceivers, which further motivates the development of channel estimators for full-duplex systems. A novel cancellation method is proposed in [14] by adding a cancelling signal to the original signal.

In addition to the SI channel information for SI cancellation, intended channel knowledge is an important prerequisite for signal detection. Motivated by this fact, channel estimation has been the subject of intense research. In the case of data-aided transmissions, training-based techniques can be applied [15, 16]. However, the amount of training increases dramatically with the number of antennas and channel order. Blind approaches have been proposed as more bandwidth efficient techniques [17, 18] where subspace methods, initially presented in [19], have a great potential. By decomposing the covariance matrix of the received signal, subspace methods exploit the orthogonality between the noise and the signal subspaces in the observation space to express the channel coefficients as a linear combination of a basis of the signal subspace. Although previous researches have shown the potential of this procedure to give an accurate estimate of the channel, it remains of limited practical interest. Actually, considering that the noise subspace needs to be nondegenerated, it is legitimate to wonder how we can satisfy this condition. Previous works rely on oversampling of the received signal or using more receive antennas than transmit antennas [20, 21]. However, such solutions increase the receiver cost and need additional hardware. Moreover, they may result in correlated noise which makes the subspace technique inappropriate. A maximum likelihood estimator was presented in [22] by exploiting the pilots in the intended signal.

In the full-duplex context, the transmitter impairments, including power amplifier (PA) nonlinearity and IQ mixer imbalance, become limiting factors and need to be reduced to properly detect the intended signal. In practice, the inband image resulting from the IQ mixer in mobile user is about 28 dB lower than the direct signal [23]. In the presence of strong SI of about 50 dB higher than the intended signal, this IQ image represents additional interference for the intended signal. The effects of transceiver impairments are illustrated in detail in [3, 24]. Due to the importance of the nonlinearities, a digital cancellation procedure has been proposed to reduce the effects of the PA in [25] by estimating the nonlinear coefficients of the PA and another algorithm has been proposed to deal with the IQ mixer imbalance [26]. However, there is no discussion about the intended signal in the existing literature, which limits the estimation performance if it is considered as additive noise.

In this work, we incorporate the intended signal in the estimation process. We also take into account the transmitter impairments when modelling the SI signal. For realistic multipath propagation channels, we need to estimate the SI channel, the intended channel and the distorted SI. And noting that the intended signal is unknown, we propose to use a novel subspace method to efficiently estimate the different parameters. Since the received signal consists of the SI and intended signals, the dimension of the signal subspace in full-duplex operation is at least twice that in traditional half-duplex operation [5, 27]. Thus an essential shortcoming of the existing subspace-based technique is that it can be applied only when the number of receive antennas is larger than the number of transmit antennas. In the following, we circumvent this condition and develop a subspace-based algorithm suitable for MIMO full-duplex systems with larger or equal numbers of transmit and receive antennas. We exploit both the covariance and pseudo-covariance matrices of the received signal to effectively increase the dimension of the observation space while keeping the dimension of the signal subspace unchanged. The joint processing of the received signal and its complex conjugates has been used in many works to improve the detection performance on various systems [28, 29]. Also, in an entirely different context, the improper property of the received signal was first exploited for channel identification in [30] to obtain a virtual SIMO model from a SISO one. Preliminary results can be found in [31] for real-valued symbols to enable the application of widely linear processing techniques, but entail a loss in spectral efficiency compared to complex-valued symbols. We propose in this paper a method to use the widely linear processing to complex symbols by forcing the transmit signal to be improper. We justify the advocated time domain approach and compare its performances to a frequency domain approach and we generalize the PA model to any nonlinearity order. In practice, we cannot blindly recover the channel coefficients since an ambiguity term always appears in the final estimate [5]. This ambiguity is resolved using a sequence of pilot symbols, considerably shorter than needed in training-based techniques. In the following, we propose a joint data detection and estimation of the ambiguity term to considerably reduce the length of the pilot sequence. We show through simulation that just one pilot symbol is sufficient to perfectly estimate the channel.

The paper is organized as follows. In Section 2, the full-duplex system model is presented. The subspace-based channel estimation is described in Section 3. In Section 4, we describe the joint decoding and ambiguity removal procedure. Illustrative simulation results are given in Section 5 and Section 6 presents the conclusion.

Notations commonly used in this paper are presented. Subscripts (·)^∗^, (·)^*T*^, and (·)^*H*^ refer to conjugate, transpose and conjugate transpose for matrices or vectors, respectively. For a given vector ***x***, diag(***x***) returns a diagonal matrix whose diagonal elements are the entries of ***x***. rank(***M***) returns the rank of a given matrix ***M***, det(***M***) returns the determinant of ***M*** and vect(***M***) stacks the columns of ***M*** into one vector. The operator ⊗ refers to the Kronecker product of two matrices. ℜ(·) and *I*(·) return the real and imaginary parts of complex numbers. *E*(·) denotes the mathematical expectation. ||·||_2_ returns the Euclidean norm of a vector. ***I***_*p*_ refers to the *p*×*p* identity matrix and ***1***_*p*_ the *p*×1 vector with 1 at all elements. A term accented by a hat, $\widehat x$, means an estimate of *x*.

## Full-duplex MIMO system model

**Fig. 1:**
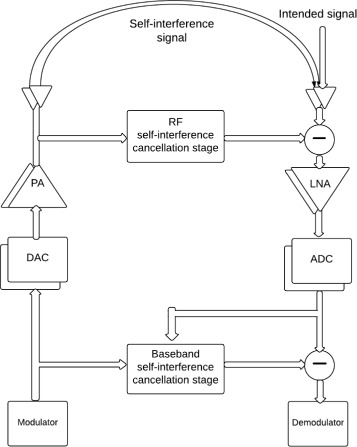
Simplified block diagram of the full-duplex transceiver with RF and baseband SI cancellation stages

Consider two transceivers communicating in a full-duplex fashion. The simultaneous transmission and reception creates self-interference (SI) to be cancelled before the demodulation process. The SI signal is first suppressed at RF, prior to the low-noise amplifier (LNA) and analog-to-digital converter (ADC) to avoid overloading/saturation of these components [2, 3, 32]. In [5], we proposed an efficient compressed-sensing (CS)-based algorithm for the RF SI cancellation stage. In this work, we concentrate on the development of subspace-based algorithm to jointly estimate the SI and intended channels and the nonlinear distortions for the baseband SI cancellation stage of a full-duplex MIMO transceiver with arbitrary numbers of transmit and receive antennas. The output signal of the RF SI cancellation stage consists of the residual SI, the intended signal received from the other transceiver and the additive thermal noise. Figure 1 shows a simplified block diagram of a MIMO transceiver. The residual SI can be further suppressed at the baseband after ADC using digital signal processing (DSP). The advantage of working in the digital domain, as compared to RF, is that sophisticated DSP methods can be handled. Both transceivers are equipped with *N*_*t*_ transmitting antennas and *N*_*r*_ receiving antennas. At transmitting antenna *q*, a group of *N* data symbols ***X***_*q*_=[ *X*_*q*_(0),…, *X*_*q*_(*N*−1)]^*T*^ is first modulated by the IFFT matrix to form an OFDM block, then the time domain vector ***x***_*q*_=[ *x*_*q*_(0),…, *x*_*q*_(*N*−1)]^*T*^ is extended by the cyclic prefix of length^1^ *N*_*cp*_ and the resulting vector is sent sequentially. In the transmit stream *q*, the complex signal *x*_*q*_(*t*) after the digital-to-analog conversion (DAC), is passed through an imbalance IQ mixer whose output is as follows:

1 $$ x_{q}^{IQ}(t) = k_{1,q} x_{q}(t) + k_{2,q} x_{q}^{*}(t),  $$

where *k*_1,*q*_ and *k*_2,*q*_ are the responses of the IQ mixer at antenna *q* to the direct signal and the image, respectively. Then, the signal is amplified with a nonlinear PA. In the following, we model the PA response with a Hammerstein model whose response is:

2 $$\begin{array}{@{}rcl@{}} x^{PA}_{q}(t) = \left(\sum_{p=0}^{P}\alpha_{2p+1,q} x_{q}^{IQ}(t)|x_{q}^{IQ}(t)|^{2p} \right) \star f(t), \end{array} $$

where *α*_2*p*+1,*q*_, for *p*=0,…, *P*, are the nonlinearity coefficients of the PA at transmit antenna *q*, *P* is the nonlinearity order and *f*(*t*) is the memory of the PA. In (2), ⋆ denotes the convolution operator. The transmitted signal is coupled to produce SI in the receiver. Considering multipath channels, the received signal at antenna *r* is as follows:

3 $$ y_{r}^{ant}(t) \! = \! \sum_{q=1}^{N_{t}} h^{c}_{r,q}(t) \star x_{q}^{PA}(t) \! + \! \sum_{q=1}^{N_{t}} h^{s}_{r,q}(t) \star s_{q}(t) \! + \! w_{th,r}(t),  $$

where *s*_*q*_(*t*) is the transmitted signal from the *q* ^*th*^ antenna of the other intended transceiver. $h_{r,q}^{c}(t)$ is the response of the SI channel from transmitting antenna *q* to receiving antenna *r* of the same transceiver. $h_{r,q}^{s}(t)$ is the response of the intended channel from transmitting antenna *q* of the other intended transceiver to receiving antenna *r* of the same transceiver. *w*_*th,r*_(*t*) is the additive thermal noise in Rx stream *r*. To reduce the SI before the LNA and ADC, the RF cancellation stage is performed as follows:

4 $$\begin{array}{@{}rcl@{}} y_{r}^{RF}(t) = y_{r}^{ant}(t) - \sum_{q=1}^{N_{t}} \widehat h^{c}_{r,q}(t) \star x_{q}^{PA}(t), \end{array} $$

where $\widehat h^{c}_{r,q}(t)$ is a first estimate of the SI channel [1, 6]. $\widehat h^{c}_{r,q}(t)$ is used to adjust the phase, amplitude and delay of the SI to the main propagation path. To include the transmitter distortion in the RF cancellation process, the reference signal is taken from the output of the PA. This RF SI cancellation can attenuate the SI by 30 dB, as reported in practical experiments [6, 33]. Then, the received signal passes through the LNA:

5 $$ y_{r}^{LNA}(t) = k_{LNA} y_{r}^{RF}(t) + w_{LNA}(t),  $$

where *w*_*LNA*_(*t*) is the additive noise caused by the LNA and *k*_*LNA*_ is the gain of the LNA. Finally, the received signal is adjusted by the variable gain amplifier (VGA) to match the dynamic range of the ADC. For simplicity, we suppose that the linear gains *k*_1,*q*_ and *α*_1,*q*_ of the IQ mixer and PA are equal to 1. Combining (2), (3) and (5), the received samples are given by

6 $$ \begin{aligned} y_{r}(n) &= \sum_{q=1}^{N_{t}} \sum_{l=0}^{L} h^{(i)}_{r,q}(l)x_{q}^{IQ}(n-l) + \sum_{p=1}^{P} \alpha_{2p+1,q} h^{(i)}_{r,q}(l)x_{q,ip,p}(n-l)\\ &\quad+ h_{r,q}^{(s)}(l) s_{q}(n-l) + w_{r}(n), \end{aligned}  $$

where $x_{{q,ip,p}}(n)=x_{q}^{IQ}(n)|x_{q}^{IQ}(n)|^{2p}$ resulting from the cascade of IQ mismatch and PA (2*p*+1)^*rd*^ order nonlinearity and *w*_*r*_(*n*) collects the thermal noise, the LNA noise and the quantization noise. In (6), the global channel responses are given by

7 $$\begin{array}{@{}rcl@{}} h^{(i)}_{r,q}(l) & = & k_{LNA} (h^{c}_{r,q}(l) \star f(l) -\widehat h_{r,q}^{c}(l)),\\ h^{(s)}_{r,q}(l) & = & k_{LNA} h_{r,q}^{s}(l). \end{array} $$

To have a homogeneous notation, all channels are supposed to have the same order *L* and the channels of order lower than *L* are zero-padded so that the different channels have the same order and *L* still satisfies *L*<*N*_*cp*_. The received vector $\boldsymbol {y}(n)=\ [\!y_{1}(n),\dots,~y_{N_{r}}(n)]^{T}$ over the *N*_*r*_ antennas is given by

8 $$ \begin{aligned} \boldsymbol{y}(n) &= \sum_{q=1}^{N_{t}} \sum_{l=0}^{L} \boldsymbol{h}_{q}^{(i)}(l) x_{q}^{IQ}(n-l) + \sum_{p=1}^{P} \alpha_{2p+1,q} \boldsymbol{h}_{q}^{(i)}(l) x_{q,ip,p}(n-l)\\ &\quad+ \boldsymbol{h}_{q}^{(s)}(l) s_{q}(n-l) + \boldsymbol{w}(n), \end{aligned}  $$

where

9 $$\begin{array}{@{}rcl@{}} \boldsymbol{h}^{(i)}_{q}(l) & = & [\!h^{(i)}_{1,q}(l),~ h^{(i)}_{2,q}(l),\dots,~ h^{(i)}_{N_{r},q}(l)]^{T},\\ \boldsymbol{h}^{(s)}_{q}(l) & = & [\!h^{(s)}_{1,q}(l),~ h^{(s)}_{2,q}(l),\dots,~ h^{(s)}_{N_{r},q}(l)]^{T}, \end{array} $$

for *l*=0, 1,…, *L* and $\boldsymbol {w}(n) =\ [w_{1}(n),~w_{2}(n),\dots, w_{N_{r}}(n)]^{T}$. For a more compact representation, we gather the transmitted signals from the *N*_*t*_ antennas to obtain

10 $$ \boldsymbol{y}(n) = \sum_{l=0}^{L} \boldsymbol{H}^{(i)}(l) \boldsymbol{x}(n-l) + \boldsymbol{H}^{(s)}(l) \boldsymbol{s}(n-l) + \boldsymbol{w}(n),  $$

where the *N*_*r*_×*N*_*t*_ matrices ***H*** ^(*i*)^(*l*) and ***H*** ^(*s*)^(*l*) are given by

11 $$\begin{array}{@{}rcl@{}} \boldsymbol{H}^{(i)}(l) = [\!\boldsymbol{h}^{(i)}_{1}(l),~\boldsymbol{h}^{(i)}_{2}(l),\dots,~\boldsymbol{h}^{(i)}_{N_{t}}(l)], \\ \boldsymbol{H}^{(s)}(l) = [\!\boldsymbol{h}^{(s)}_{1}(l),~\boldsymbol{h}^{(s)}_{2}(l),\dots,~\boldsymbol{h}^{(s)}_{N_{t}}(l)], \end{array} $$

for *l*=0,…, *L* and

12 $$ \begin{aligned} \boldsymbol{x}_{i}(n) & =\ [x_{1}(n),~x_{2}(n),\dots,~x_{N_{t}}(n)]^{T}, \\ \boldsymbol{x}_{dist}(n) & = \left[k_{2,1}x_{1}^{*}(n)+\sum_{p=1}^{P} \alpha_{2p+1,1}x_{1,ip,p}(n),\dots,~k_{2,N_{t}}x_{N_{t}}^{*}(n)\right.\\&\left.\qquad+\sum_{p=1}^{P} \alpha_{2p+1,N_{t}}x_{N_{t},ip,p}(n)\right]^{T}, \\ \boldsymbol{x}(n) & = \boldsymbol{x}_{i}(n) + \boldsymbol{x}_{dist}(n),\\ \boldsymbol{s}(n) & = [\!s_{1}(n),~s_{2}(n),\dots,~s_{N_{t}}(n)]^{T}. \end{aligned}  $$

We then group the channel matrices ***H*** ^(*i*)^(*l*) and ***H*** ^(*s*)^(*l*) in one *N*_*r*_×2*N*_*t*_ matrix ***H***(*l*)= [***H*** ^(*i*)^(*l*), ***H*** ^(*s*)^(*l*)] and gather all the channel coefficients in the following *N*_*r*_ *M*×2*N*_*t*_ *N* block Toeplitz matrix:

13 $$\begin{array}{@{}rcl@{}} \boldsymbol{H} = \left(\begin{array}{lllll} \boldsymbol{H}(0) & \boldsymbol{0}~\dots & \boldsymbol{0}~\boldsymbol{H}(L) & \dots & \boldsymbol{H}(1) \\ \boldsymbol{H}(1) & \boldsymbol{H}(0) & & \ddots & \vdots \\ \vdots & \boldsymbol{H}(1) & \ddots & & \boldsymbol{H}(L) \\ \boldsymbol{H}(L) & \vdots & & \ddots & \boldsymbol{0} \\ & \boldsymbol{H}(L) & & & \boldsymbol{H}(0) \\ \boldsymbol{0} & & \ddots & & \boldsymbol{H}(1) \\ \vdots & & & \ddots & \vdots \\ \boldsymbol{0} & \dots & \boldsymbol{0} & & \boldsymbol{H}(L) \end{array}\right). \end{array} $$

The received OFDM block on the *N*_*r*_ antennas is:

14 $$ \boldsymbol{y} =\ [\!\boldsymbol{y}^{T}(0),~\boldsymbol{y}^{T}(1),\dots,~\boldsymbol{y}^{T}(M-1)]^{T} = \boldsymbol{H} \boldsymbol{u} + \boldsymbol{w},  $$

where *M*=*N*+*L*, the 2*N*_*t*_ *N*×1 data vector ***u*** is given by

15 $$ \boldsymbol{u} =\ [\!\boldsymbol{x}^{T}(0),~\boldsymbol{s}^{T}(0),\dots,~\boldsymbol{x}^{T}(N-1),~\boldsymbol{s}^{T}(N-1)]^{T},  $$

and

16 $$ \boldsymbol{w} =\ [\!\boldsymbol{w}^{T}(0),~\boldsymbol{w}^{T}(1),\dots,~\boldsymbol{w}^{T}(M-1)]^{T}.  $$

For multi-block transmission, the received vector in (14) is indexed by the block number *t*, i.e., ***y***_*t*_. For convenience, we omit this indexation and we will consider later a given number of transmitted blocks to compute the covariance matrix of the received vector.

## Subspace-based channel estimator

We propose to apply a subspace-based algorithm to jointly estimate the SI and intended channel coefficients along with the nonlinear coefficients. Subspace methods rely on the orthogonality property between the signal and noise subspaces. These two subspaces are obtained from eigendecomposition of the covariance matrix of the received signal ***y***. Denoting by ***R***_*u*_, the covariance of ***u***, the covariance matrix ***R***_*y*_ of the received vector ***y*** is given by

17 $$ \boldsymbol{R}_{y} = \boldsymbol{H} \boldsymbol{R}_{u} \boldsymbol{H}^{H} +\sigma^{2} \boldsymbol{I}_{MN_{r}},  $$

as long as the signal samples are uncorrelated from the noise samples^2^.

The signal subspace is spanned by the columns of the matrix ***H***. Noting that the columns of ***H*** are, by construction, linearly independent as soon as there exists an *l*∈ [ 0, *L*] such that ***H***(*l*) is full rank^3^, the matrix ***H*** is a full-rank matrix. Therefore, the dimension of the signal subspace is 2*NN*_*t*_. It follows that, to obtain a nondegenerate noise subspace, its dimension *N*_*r*_ *M*−2*N*_*t*_ *N* should be larger than zero, and thus, the number of receiving antennas should be larger than the number of transmitting antennas to make the subspace method work, and in [5], we developed the linear subspace algorithm for this setting. In the following, we will develop the subspace-based algorithm for general numbers of transmit and receive antennas. When *N*_*t*_=*N*_*r*_, the matrix ***R***_*y*_ cannot be directly used to find the noise subspace. As an alternative different approach, we consider the augmented received vector as

18 $$\begin{array}{@{}rcl@{}} \widetilde{\boldsymbol{y}} = \left(\begin{array}{l} \boldsymbol{y} \\ \boldsymbol{y}^{*} \end{array}\right) = \left(\begin{array}{ll} \boldsymbol{H} & \mathbf{0} \\ \mathbf{0} & \boldsymbol{H}^{*} \end{array}\right) \left(\begin{array}{l} \boldsymbol{u} \\ \boldsymbol{u}^{*} \end{array}\right) + \left(\begin{array}{l} \boldsymbol{w} \\ \boldsymbol{w}^{*} \end{array}\right). \end{array} $$

The use of the augmented received vector is usually referred as widely linear processing. In this case, the augmented covariance matrix $\boldsymbol {R}_{\widetilde y}$ of $\widetilde {\boldsymbol {y}}$ has the following structure:

19 $$\begin{array}{@{}rcl@{}} \boldsymbol{R}_{\widetilde y} = \widetilde{\boldsymbol{H}} \boldsymbol{R}_{\widetilde u} \widetilde{\boldsymbol{H}}^{H} + \sigma^{2} \boldsymbol{I}_{2MN_{r}}, \end{array} $$

where $\boldsymbol {R}_{\widetilde u}$ denotes the covariance matrix of the augmented transmit signal $\widetilde {\boldsymbol {u}} = \left (\begin {array}{l} \boldsymbol {u} \\ \boldsymbol {u}^{*} \end {array}\right)$ and

20 $$ \widetilde{\boldsymbol{H}} = \left(\begin{array}{ll} \boldsymbol{H} & \mathbf{0} \\ \mathbf{0} & \boldsymbol{H}^{*} \end{array}\right).  $$

It is worth mentioning that the proper noise has a vanishing pseudo-covariance [34]. The main purpose of using the extended received signal is to increase the dimension of the received signal and thus avoid the degenerate noise subspace. Hence, the subspace identification procedure can be derived only if the signal part covariance matrix, given by $\widetilde {\boldsymbol {H}} \boldsymbol {R}_{\widetilde u} \widetilde {\boldsymbol {H}}^{H}$, of the covariance matrix $\boldsymbol {R}_{\widetilde y}$ is singular. It results that $d_{s} = \text {rank}(\widetilde {\boldsymbol {H}} \boldsymbol {R}_{\widetilde u} \widetilde {\boldsymbol {H}}^{H}) < 2MN_{r}$. In this case, the signal is confined in a *d*_*s*_-dimensional subspace and the remaining noise subspace is with dimension 2*MN*_*r*_−*d*_*s*_. Singularity of $\boldsymbol {R}_{\widetilde u}$ is a necessary condition to obtain a nondegenerate noise subspace. Actually, noting that $\widetilde {\boldsymbol {H}}$ is full rank, nonsingular $\boldsymbol {R}_{\widetilde u}$ results in $\text {rank}(\widetilde {\boldsymbol {H}} \boldsymbol {R}_{\widetilde u} \widetilde {\boldsymbol {H}}^{H}) = 2MN_{r}$, and thus, the matrix $\widetilde {\boldsymbol {H}} \boldsymbol {R}_{\widetilde u} \widetilde {\boldsymbol {H}}^{H}$ spans all the observation space. On the other hand, since the matrix $\widetilde {\boldsymbol {H}}$ is a tall matrix, singularity of $\boldsymbol {R}_{\widetilde u}$ is not a sufficient condition to guarantee the singularity of $\widetilde {\boldsymbol {H}} \boldsymbol {R}_{\widetilde u} \widetilde {\boldsymbol {H}}^{H}$.

The matrix $\boldsymbol {R}_{\widetilde u}$ can be expressed in a block form in terms of the covariance matrix of ***u***, ***R***_*u*_=*E*(***u*** ***u*** ^*H*^), the pseudo-covariance matrix ***C***_*u*_=*E*(***u*** ***u*** ^*T*^) and their complex conjugates as

21 $$ \boldsymbol{R}_{\widetilde u} = \left(\begin{array}{ll} \boldsymbol{R}_{u} & \boldsymbol{C}_{u} \\ \boldsymbol{C}_{u}^{*} & \boldsymbol{R}_{u}^{*} \end{array}\right).  $$

In the following, we distinguish two cases of real and complex modulated symbols.

For real modulated symbols, it can be shown that $\boldsymbol {R}_{\widetilde u} = \alpha ^{2} \boldsymbol {M} \otimes \boldsymbol {I}_{2N_{t}}$ with the 2*N*×2*N* matrix ***M*** having the following form: 
22


From (22), we note that each column of ***M*** appears exactly two times (the first column of ***M*** is the same as the (*N*+1)^*th*^ column, and the *i* ^*th*^ column of ***M*** is the same as the (2*N*−*i*+2)^*th*^ column, for *i*=2,…, *N*). Therefore, the matrix ***M*** has exactly *N*-independent columns and thus its rank is *N*. It follows that the rank of $\boldsymbol {R}_{\widetilde u}$ is 2*NN*_*t*_. In Appendix 1, we show that $\boldsymbol {R}_{\widetilde u}$ has zero eigenvalue with multiplicity 2*NN*_*t*_ and 2*α* ^2^ also with multiplicity 2*NN*_*t*_. Then, the matrix $\boldsymbol {R}_{\widetilde u}$ is decomposed as ***U*** ***D*** ***U*** ^*H*^ where ***D*** is the 4*NN*_*t*_×4*NN*_*t*_ diagonal matrix with zeroes in the first 2*NN*_*t*_ diagonal elements and 2*α* ^2^ in the last 2*NN*_*t*_ diagonal elements and ***U*** is an orthogonal matrix whose columns are the corresponding eigenvectors of $\boldsymbol {R}_{\widetilde u}$.

For complex symbols, the pseudo-covariance matrix ***C***_*u*_ is generally equal to the zero matrix, which makes the matrix $\boldsymbol {R}_{\widetilde u}$ of full rank. To avoid this problem, we apply a simple precoding at the input of the IFFT. It transforms the data symbol ***X***_*q*_ to

23 $$\begin{array}{@{}rcl@{}} \widetilde{\boldsymbol{X}}_{q} = \boldsymbol{P} \boldsymbol{X}_{q} + \boldsymbol{Q} \boldsymbol{X}_{q}^{*}. \end{array} $$

where ***P*** and ***Q*** are two matrices. By combining the data symbol ***X***_*q*_ and its complex conjugate, we force the pseudo-covariance matrix to be different from zero. Appendix 2 gives a detailed discussion about the choice of the matrices ***P*** and ***Q*** so that the covariance matrix $\boldsymbol {R}_{\widetilde u}$ has rank 2*NN*_*t*_ and can be decomposed as ***U*** ***D*** ***U*** ^*H*^ with ***D*** as the 4*NN*_*t*_×4*NN*_*t*_ diagonal matrix with zeroes in the first 2*NN*_*t*_ diagonal elements.

The noise subspace is the span of the *p*=2*MN*_*r*_−2*NN*_*t*_ eigenvectors of $\boldsymbol {R}_{\widetilde y}$ corresponding to the smallest eigenvalue *σ* ^2^, and the columns of $\widetilde {\boldsymbol {H}} \boldsymbol {R}_{\widetilde u} \widetilde {\boldsymbol {H}}^{H}$ belong to the signal subspace. Due to the orthogonality between the signal and the noise subspaces, each column of $\widetilde {\boldsymbol {H}} \boldsymbol {R}_{\widetilde u} \widetilde {\boldsymbol {H}}^{H}$ is orthogonal to any vector in the noise subspace. Let $\{\boldsymbol {\nu }_{i}\}_{i=1}^{p}$ denote the *p* co-orthogonal eigenvectors corresponding to the smallest eigenvalue of $\boldsymbol {R}_{\widetilde y}$. Then we have the following set of equations:

24 $$ \boldsymbol{\nu}_{i}^{H} \widetilde{\boldsymbol{H}} \boldsymbol{R}_{\widetilde u} \widetilde{\boldsymbol{H}}^{H} = \mathbf{0},~i=1,~2,\dots,~p.  $$

From (24), we conclude that ***ν***_*i*_ spans the left null space of $ \widetilde {\boldsymbol {H}} \boldsymbol {R}_{\widetilde u} \widetilde {\boldsymbol {H}}^{H}$. For convenience, ***U*** is written as a block of 4 2*NN*_*t*_×2*NN*_*t*_ matrices:

25 $$ \boldsymbol{U} = \left(\begin{array}{cccccc} \boldsymbol{U}_{1} & \boldsymbol{U}_{2} \\ \boldsymbol{U}_{3} & \boldsymbol{U}_{4} \end{array} \right),  $$

where the columns of $[\boldsymbol {U}_{1}^{T},~\boldsymbol {U}_{3}^{T}]^{T}$ are the eigenvectors of $\boldsymbol {R}_{\widetilde u}$ corresponding to the eigenvalue zero and the columns of $[\boldsymbol {U}_{2}^{T},~\boldsymbol {U}_{4}^{T}]^{T}$ are the other eigenvectors. Then, taking into account the eigenvalue decomposition of $\boldsymbol {R}_{\widetilde u}$, the set of equations in (24) are equivalent to

26 $$\begin{array}{@{}rcl@{}} \boldsymbol{\nu}_{i}^{H} \left(\begin{array}{c} \boldsymbol{H} \boldsymbol{U}_{2}\\ \boldsymbol{H}^{*} \boldsymbol{U}_{4} \end{array} \right) = \mathbf{0},~i=1,~2,\dots,~p. \end{array} $$

By dividing ***ν***_*i*_ into two *MN*_*r*_×1 vectors, i.e., $\boldsymbol {\nu }_{i} = [\boldsymbol {\nu }_{i,1}^{T},~\boldsymbol {\nu }_{i,2}^{T}]^{T}$, (26) is rewritten as

27 $$ \boldsymbol{\nu}_{i,1}^{H} \boldsymbol{H} \boldsymbol{U}_{2} + \boldsymbol{\nu}_{i,2}^{H} \boldsymbol{H}^{*} \boldsymbol{U}_{4} = \mathbf{0},  $$

for *i*=1, 2,…,*p*. The matrix ***H*** is completely defined by the set of matrices ***H***(*l*), for *l*=0, 1,…, *L*. Therefore, the specific structure of ***H*** should be taken into consideration when solving the equations in (27) to obtain a more accurate estimate of the channels. To that end, we divide the two vectors ***ν***_*i*,1_ and ***ν***_*i*,2_ as follows:

28 $$ \begin{aligned} \boldsymbol{\nu}_{i,j} &= \left[\boldsymbol{\nu}_{i,j}^{T}(M),~\boldsymbol{\nu}_{i,j}^{T}(M-1),\dots,~\boldsymbol{\nu}_{i,j}^{T}(1)\right]^{T},\\ j&=1,~2,~i=1,~2,\dots,~p, \end{aligned}  $$

where each ***ν***_*i,j*_(*n*), for *n*=1, 2,…, *M*, is a *N*_*r*_×1 vector. From (13) and (28), each term $\boldsymbol {\nu }_{i,1}^{H} \boldsymbol {H}$ in (27) is rewritten as

29 $$ \begin{aligned} &\sum_{l=0}^{L} \boldsymbol{\nu}_{i,1}^{H}(n+L-l) \boldsymbol{H}(l) + \sum_{l=n}^{L} \boldsymbol{\nu}_{i,1}^{H}(M-l+n) \boldsymbol{H}(l),\\&\quad\text{for}~n=1,~\dots,~L,\\ &\sum_{l=0}^{L} \boldsymbol{\nu}_{i,1}^{H}(n+L-l) \boldsymbol{H}(l), \text{for}~n=L+1,\dots,~M, \end{aligned}  $$

and $\boldsymbol {\nu }_{i,2}^{H} \boldsymbol {H}^{*}$ can also be partitioned in the same manner. By introducing $\boldsymbol {\check {h}}(l) = \text {vect}(\boldsymbol {H}(l))$ and $\boldsymbol {V}_{i,j}(n) = \boldsymbol {I}_{2N_{t}} \otimes \boldsymbol {\nu }_{i,j}^{H}(n)$, for *i*=1,…, *p* and *j*=1, 2, it is easy to verify that $\boldsymbol {\nu }_{i,j}^{H}(n) \boldsymbol {H}(l) = \boldsymbol {\check h}^{T}(l) \boldsymbol {V}_{i,j}^{T}(n)$. Let us denote the 2*NN*_*t*_×2*N*_*t*_ *N*_*r*_(*L*+1) matrices ***V***_*i,j*_, for *j*=1, 2, as

30 $$ { \begin{aligned} \boldsymbol{V}_{i,j} &= \left(\begin{array}{llll} \boldsymbol{V}_{i,j}(L+1) & \boldsymbol{V}_{i,j}(L) & \ldots & \boldsymbol{V}_{i,j}(1) \\ \boldsymbol{V}_{i,j}(L+2) & \boldsymbol{V}_{i,j}(L+1) & \ldots & \boldsymbol{V}_{i,j}(2) \\ \boldsymbol{V}_{i,j}(L+3) & \boldsymbol{V}_{i,j}(L+2) & \ldots & \boldsymbol{V}_{i,j}(3) \\ \vdots & \vdots & \vdots & \vdots \\ \boldsymbol{V}_{i,j}(N+L) & \boldsymbol{V}_{i,j}(N+L-1)& \ldots & \boldsymbol{V}_{i,j}(N) \\ \end{array}\right)\\ &\quad+ \left(\begin{array}{llll} \mathbf{0} & \boldsymbol{V}_{i,j}(N+L) & \ldots & \boldsymbol{V}_{i,j}(N+1) \\ & & \ddots & \vdots \\ \vdots& & & \boldsymbol{V}_{i,j}(N+L) \\ \vdots& & & \mathbf{0} \\ & & & \vdots \\ \mathbf{0} & & & \mathbf{0} \\ \end{array}\right), \end{aligned}}  $$

and $\boldsymbol {\check {h}} = [\boldsymbol {\check h}^{T}(0),~\boldsymbol {\check h}^{T}(1),\dots,~\boldsymbol {\check h}^{T}(L)]^{T}$. Then, using the previous notations, (27) is rearranged to obtain

31 $$\begin{array}{@{}rcl@{}} \boldsymbol{\check h}^{T} \boldsymbol{V}_{i,1}^{T} \boldsymbol{U}_{2} + \boldsymbol{\check h}^{H} \boldsymbol{V}_{i,2}^{T} \boldsymbol{U}_{4} = \mathbf{0}, \end{array} $$

or, by taking the transpose of the previous equation:

32 $$\begin{array}{@{}rcl@{}} \boldsymbol{U}_{2}^{T} \boldsymbol{V}_{i,1} \boldsymbol{\check h} + \boldsymbol{U}_{4}^{T} \boldsymbol{V}_{i,2} \boldsymbol{\check h}^{*} = \mathbf{0}, \end{array} $$

for *i*=1, 2,…, *p*. Note that the difference between (27) and (32) is that (32) takes into account the Toeplitz blocks structure of ***H***. Now, collecting all the previous equations, we obtain

33 $$ \boldsymbol{\Theta}_{1} \boldsymbol{\check h} +\boldsymbol{\Theta}_{2} \boldsymbol{\check h}^{*} = \mathbf{0},  $$

where

34 $$ { \begin{aligned} \boldsymbol{\Theta}_{1} & = \left[\left(\boldsymbol{U}_{2}^{T} \boldsymbol{V}_{1,1}\right)^{T},~\left(\boldsymbol{U}_{2}^{T} \boldsymbol{V}_{2,1}\right)^{T},\dots,~\left(\boldsymbol{U}_{2}^{T} \boldsymbol{V}_{p,1}\right)^{T}\right]^{T},\\ \boldsymbol{\Theta}_{2} & = \left[\left(\boldsymbol{U}_{4}^{T} \boldsymbol{V}_{1,2}\right)^{T},~\left(\boldsymbol{U}_{4}^{T} \boldsymbol{V}_{2,2}\right)^{T},\dots,~\left(\boldsymbol{U}_{4}^{T} \boldsymbol{V}_{p,2}\right)^{T}\right]^{T}. \end{aligned}}  $$

Separating the real and imaginary parts of (33), we have

35 $$\begin{array}{*{20}l} \underbrace{\left(\begin{array}{ll} \Re(\boldsymbol{\Theta}_{1}+\boldsymbol{\Theta}_{2}) & \Im(-\boldsymbol{\Theta}_{1}+\boldsymbol{\Theta}_{2})\\ \Im(\boldsymbol{\Theta}_{1}+\boldsymbol{\Theta}_{2}) & \Re(\boldsymbol{\Theta}_{1}-\boldsymbol{\Theta}_{2}) \end{array}\right)}_{\boldsymbol{\overline \Theta}} \underbrace{\left(\begin{array}{l} \Re(\boldsymbol{\check h}) \\ \Im(\boldsymbol{\check h}) \end{array}\right)}_{\boldsymbol{\overline h}} = \mathbf{0}. \end{array} $$

From (35), the vector $\boldsymbol {\overline h}$ belongs to the right null space of $\boldsymbol {\overline \Theta }$. In practice, $\boldsymbol {\overline h}$ is a linear combination of the 4*N*_*t*_ *N*_*r*_ right singular vectors of the matrix $\boldsymbol {\overline \Theta }$, denoted by ***β***_*i*_, which are equal to the eigenvector of the Gramian $\overline {\boldsymbol {\Theta }}\overline {\boldsymbol {\Theta }}^{H}$ corresponding to the zero eigenvalue. Therefore, an estimate of $\overline {\boldsymbol {h}}$ is given by

36 $$ \widehat{\overline{\boldsymbol{h}}} = \overline{\boldsymbol{\Phi}} \boldsymbol{c},  $$

where $\overline {\boldsymbol {\Phi }}=[\boldsymbol {\beta }_{1},~\boldsymbol {\beta }_{2},\dots,~\boldsymbol {\beta }_{4N_{t}N_{r}}]$, and the 4*N*_*t*_ *N*_*r*_×1 vector ***c*** represents the ambiguity term to be estimated. The complex channel vector can also be obtained as

37 $$ \widehat{\boldsymbol{\check h}} = \boldsymbol{\Phi} \boldsymbol{c},  $$

where ***Φ*** is obtained by combining the lines of $\overline {\boldsymbol {\Phi }}$ in the following way:

38 $$\begin{array}{@{}rcl@{}} \overline{\boldsymbol{\Phi}} = \left(\begin{array}{l} \overline{\boldsymbol{\Phi}}_{real} \\ \overline{\boldsymbol{\Phi}}_{imag} \end{array}\right) \rightarrow \boldsymbol{\Phi} = \overline{\boldsymbol{\Phi}}_{real} + j\overline{\boldsymbol{\Phi}}_{imag}, \end{array} $$

and *j* is the complex number satisfying *j* ^2^=−1.

We mention that the matrices ***U***_***2***_ and ***U***_***4***_ do not depend on the received signal and can be computed offline prior to the transmission. It is also seen that the overestimated channel order *L* does not affect the estimation process. This is a common property with other subspace-based estimators [17].

## Resolving the ambiguity term

As mentioned above, the subspace that contains the channels is obtained and the ambiguity term needs to be estimated to extract the exact coefficients. Different approaches can be applied to solve the ambiguity term ***c***. To do so, we highlight the contribution of ***c*** on the received vector ***y***. First, we separate the matrix ***Φ*** in two *N*_*t*_ *N*_*r*_(*L*+1)×4*N*_*t*_ *N*_*r*_ matrices ***Φ***_*i*_ and ***Φ***_*s*_ which contribute in the SI and intended channels, respectively (i.e., $\boldsymbol {\check h}^{(i)} = \boldsymbol {\Phi }_{i} \boldsymbol {c}$ and $\boldsymbol {\check h}^{(s)} = \boldsymbol {\Phi }_{s} \boldsymbol {c}\big)$. By rearranging the elements of ***Φ***_*i*_ as

39 $$ \boldsymbol{\Phi}_{i} \,=\, \left(\begin{array}{l} \boldsymbol{\Phi}_{i,1}(0) \\ \boldsymbol{\Phi}_{i,2}(0) \\ \vdots \\ \boldsymbol{\Phi}_{i,N_{t}}(0) \\ \vdots \\ \boldsymbol{\Phi}_{i,1}(L) \\ \boldsymbol{\Phi}_{i,2}(L) \\ \vdots \\ \boldsymbol{\Phi}_{i,N_{t}}(L) \\ \end{array}\right) \rightarrow \boldsymbol{\check \Phi}_{i} \,=\, \left(\begin{array}{lll} \boldsymbol{\Phi}_{i,1}(0) & \dots & \boldsymbol{\Phi}_{i,N_{t}}(0) \\ \boldsymbol{\Phi}_{i,1}(1) & \dots & \boldsymbol{\Phi}_{i,N_{t}}(1) \\ \vdots & & \vdots \\ \boldsymbol{\Phi}_{i,1}(L) & \dots & \boldsymbol{\Phi}_{i,N_{t}}(L) \\ \end{array}\right)\!\!,\!  $$

where each ***Φ***_*i,q*_(*l*) is a *N*_*r*_×4*N*_*t*_ *N*_*r*_ matrix, $\boldsymbol {\check H}^{(i)} = [{\boldsymbol {H}^{(i)}}^{T}(0),~{\boldsymbol {H}^{(i)}}^{T}(1),\dots,~{\boldsymbol {H}^{(i)}}^{T}(L)]^{T}$ can be written as

40 $$ \boldsymbol{\check H}^{(i)} = \boldsymbol{\check \Phi}_{i} (\boldsymbol{I}_{N_{t}}\otimes \boldsymbol{c}),  $$

and $\boldsymbol {\check H}^{(s)} = [{\boldsymbol {H}^{(s)}}^{T}(0),~{\boldsymbol {H}^{(s)}}^{T}(1),\dots,~{\boldsymbol {H}^{(s)}}^{T}(L)]^{T}$ can be also written as $\boldsymbol {\check H}^{(s)} = \boldsymbol {\check \Phi }_{s} (\boldsymbol {I}_{N_{t}} \otimes \boldsymbol {c}),$ where $\boldsymbol {\check \Phi }_{s}$ is defined in the same way as $\boldsymbol {\check \Phi }_{i}$. $\boldsymbol {\check H}^{(i)}$ and $\boldsymbol {\check \Phi }_{i}$ are used to build the matrices ***H*** ^(*i*)^ and ***Ψ***_*i*_, respectively, having the same block structure as ***H*** in (13).

Next, we define the diagonal matrices ***K*** and ***A***_*p*_ whose diagonal elements are $\boldsymbol {k} = [k_{2,1},\dots,~k_{2,N_{t}}]^{T}$ and $\boldsymbol {\alpha }_{p} = [\alpha _{2p+1,1},\dots,~\alpha _{2p+1,N_{t}}]^{T}$, respectively, and we denote by $\boldsymbol {x}_{ip,p}(n) = [ x_{1,ip,p}(n),\dots,~ x_{N_{t},ip,p}(n)]^{T}$, and $\boldsymbol {x}_{ip,p} = [\boldsymbol {x}_{ip,p}^{T}(0),\dots,~\boldsymbol {x}_{ip,p}^{T}(N-1)]^{T}$. Using the previous notations and by developing $\boldsymbol {x} = \boldsymbol {x}_{i} + (\boldsymbol {I}_{N} \otimes \boldsymbol {K}) \boldsymbol {x}^{*}_{i} + \sum _{p=1}^{P} (\boldsymbol {I}_{N} \otimes \boldsymbol {A}_{p}) \boldsymbol {x}_{ip,p}$ in terms of the transmitter impairments, one can express the received signal in (14) as

41 $$ \begin{aligned} \boldsymbol{y} & = \underbrace{\boldsymbol{\Psi}_{i} (\boldsymbol{I}_{NN_{t}} \otimes \boldsymbol{c})}_{\boldsymbol{H}^{(i)}} \boldsymbol{x} + \underbrace{\boldsymbol{\Psi}_{s} (\boldsymbol{I}_{NN_{t}} \otimes \boldsymbol{c})}_{\boldsymbol{H}^{(s)}} \boldsymbol{s} + \boldsymbol{w},\\ &=\boldsymbol{\Psi}_{i} (\boldsymbol{I}_{NN_{t}} \otimes \boldsymbol{c}) \left(\boldsymbol{x}_{i} + (\boldsymbol{I}_{N} \otimes \boldsymbol{K}) \boldsymbol{x}^{*}_{i} + \sum_{p=1}^{P}(\boldsymbol{I}_{N}\otimes \boldsymbol{A}_{p}) \boldsymbol{x}_{ip,p} \right)\\ &\quad+ \boldsymbol{\Psi}_{s} (\boldsymbol{I}_{NN_{t}} \otimes \boldsymbol{c}) \boldsymbol{s} + \boldsymbol{w}, \end{aligned}  $$

where ***Ψ***_*s*_ and ***H*** ^(*s*)^ are defined in the same way as ***Ψ***_*i*_ and ***H*** ^(*i*)^, respectively, and ***s***=[***s*** ^*T*^(0),…, ***s*** ^*T*^(*N*−1)]^*T*^. After some manipulations, one can easily verify that $(\boldsymbol {I}_{NN_{t}} \otimes \boldsymbol {c}) \boldsymbol {x}_{i} = (\boldsymbol {x}_{i} \otimes \boldsymbol {I}_{4N_{t}N_{r}}) \boldsymbol {c}$ and $(\boldsymbol {I}_{NN_{t}} \otimes \boldsymbol {c}) \boldsymbol {s} = (\boldsymbol {s} \otimes \boldsymbol {I}_{4N_{t}N_{r}}) \boldsymbol {c}$. Then, the received vector in (41) is rewritten as

42 $$ \begin{aligned} \boldsymbol{y} &= \boldsymbol{\Psi}_{i} \left(\left(\boldsymbol{x}_{i} + \left(\boldsymbol{I}_{N} \otimes \boldsymbol{K} \right)\boldsymbol{x}_{i}^{*} + \sum_{p=1}^{P}\left(\boldsymbol{I}_{N} \otimes \boldsymbol{A}_{p} \right) \boldsymbol{x}_{ip,p} \right) \otimes \boldsymbol{I}_{4N_{t}N_{r}} \right) \boldsymbol{c}\\ &\quad+ \boldsymbol{\Psi}_{s} \left(\boldsymbol{s} \otimes \boldsymbol{I}_{4N_{t}N_{r}}\right) \boldsymbol{c} + \boldsymbol{w}. \end{aligned}  $$

In (42), the received vector ***y*** is expressed as a linear function of the unknown vector ***c***. This formulation makes the estimation of ***c*** more tractable. While the transmitted SI is known, the distorted parts (***I***_*N*_⊗***A***_*p*_)***x***_*ip,p*_ and $(\boldsymbol {I}_{N} \otimes \boldsymbol {K}) \boldsymbol {x}_{i}^{*}$ of the SI from the cascade of the IQ mixer and PA need to be estimated. We begin by writing the following cost function $f(\boldsymbol {c},\boldsymbol {s},\boldsymbol {K},\boldsymbol {A}_{p}) = ||\boldsymbol {y} - \boldsymbol {\Psi }_{i} ((\boldsymbol {x}_{i} + (\boldsymbol {I}_{N} \otimes \boldsymbol {K})\boldsymbol {x}_{i}^{*} + \sum _{p=1}^{P}(\boldsymbol {I}_{N} \otimes \boldsymbol {A}_{p}) \boldsymbol {x}_{ip,p}) \otimes \boldsymbol {I}_{4N_{t}N_{r}}) \boldsymbol {c} - \boldsymbol {\Psi }_{s} (\boldsymbol {s} \otimes \boldsymbol {I}_{4N_{t}N_{r}}) \boldsymbol {c}||^{2}$ depending on ***c***, ***K***, ***A***_*p*_ (for *p*=1,…, *P*) and ***s***. Given an initial estimate $\widehat {\boldsymbol {c}}$ of ***c***, the minimization of $f(\widehat {\boldsymbol {c}},\boldsymbol {s},\boldsymbol {K},\boldsymbol {A}_{p})$ with respect to ***s***, ***K*** and ***A***_*p*_ can be recast as a least square (LS) problem. Then, using the solutions $\widehat {\boldsymbol {s}}$, $\widehat {\boldsymbol {K}}$ and $\widehat {\boldsymbol {A}}_{p}$, we minimize $f(\boldsymbol {c},\widehat {\boldsymbol {s}},\widehat {\boldsymbol {K}},\widehat {\boldsymbol {A}}_{p})$ with respect to ***c***. We iterate this procedure until the estimated parameters converge. An initial estimate of ***c*** is obtained using the LS criteria as

43 $$ \widehat{\boldsymbol{c}}_{0} = (\boldsymbol{\Psi}_{i} (\boldsymbol{x}_{i} \otimes \boldsymbol{I}_{4N_{t}N_{r}}))^{\#} \boldsymbol{y},  $$

where the operator (·)^*#*^ returns the pseudo-inverse of a given matrix. At the *k* ^*th*^ iteration, the estimate $\widehat {\boldsymbol {c}}_{k-1}$ obtained at the previous iteration is used to find ***s***, ***K*** and ***A***_*p*_ (or equivalently ***k*** and ***α***_*p*_) as follows:

44 $$ {\begin{aligned} \left(\begin{array}{l} \widehat{\boldsymbol{s}}_{k} \\ \widehat{\boldsymbol{k}}_{k} \\ \widehat{\boldsymbol{\alpha}}_{1,k} \\ \vdots \\ \widehat{\boldsymbol{\alpha}}_{P,k} \end{array}\right) & = \left[\boldsymbol{\Psi}_{s} \widehat{\boldsymbol{C}}_{k-1},~\boldsymbol{\Psi}_{i} \left(\text{diag}\left(\boldsymbol{x}_{i}^{*}\right)\boldsymbol{B} \right) \right.\\ &\quad\otimes \widehat{\boldsymbol{c}}_{k-1},~\boldsymbol{\Psi}_{i} \left(\text{diag}\left(\boldsymbol{x}_{ip,1}\right) \boldsymbol{B} \right)\otimes \widehat{\boldsymbol{c}}_{k-1},\dots,\\ & \quad\left. \boldsymbol{\Psi}_{i} \left(\text{diag}\left(\boldsymbol{x}_{ip,P}\right) \boldsymbol{B} \right) \otimes \widehat{\boldsymbol{c}}_{k-1} \right]^{\#} \left(\boldsymbol{y} - \boldsymbol{\Psi}_{i} \widehat{\boldsymbol{C}}_{k-1} \boldsymbol{x}_{i} \right), \end{aligned}}  $$

where, for clarity, we introduce $\phantom {\dot {i}\!}\boldsymbol {B} = \boldsymbol {1}_{N} \otimes \boldsymbol {I}_{N_{t}}$ and $\phantom {\dot {i}\!}\widehat {\boldsymbol {C}}_{k-1} = \boldsymbol {I}_{NN_{t}} \otimes \widehat {\boldsymbol {c}}_{k-1}$ and we use the equality $\Big (\big ((\boldsymbol {I}_{N} \otimes \boldsymbol {K})\boldsymbol {x}_{i}^{*} \big) \otimes \boldsymbol {I}_{4N_{t}N_{r}} \Big) \boldsymbol {c} = \Big (\big (\text {diag}(\boldsymbol {x}_{i}^{*})\boldsymbol {B} \big) \otimes \boldsymbol {c} \Big) \boldsymbol {k}$. Then, $\widehat {\boldsymbol {s}}_{k}$ is transformed in the frequency domain and each element of the frequency domain vector is projected to its closest discrete constellation point. The obtained vector is converted back to the time domain to obtain a better estimate $\widetilde {\boldsymbol {s}}_{k}$ of ***s***.

Then, an update of ***c*** at iteration *k* is obtained as:

45 $$ \begin{aligned} \widehat{\boldsymbol{c}}_{k} &= \left(\boldsymbol{\Psi}_{i} \left(\left(\boldsymbol{x}_{i}+(\boldsymbol{I}_{N} \otimes \widehat{\boldsymbol{K}}_{k}) + \sum_{p=1}^{P}\left(\boldsymbol{I}_{N} \otimes \widehat{\boldsymbol{A}}_{p,k}\right) \boldsymbol{x}_{ip,p} \right) \otimes \boldsymbol{I}_{4N_{t}Nr} \right)\right.\\ &\quad\left.+ \boldsymbol{\Psi}_{s} \left(\widetilde{\boldsymbol{s}}_{k} \otimes \boldsymbol{I}_{4N_{t}N_{r}}\right) {\vphantom{\sum_{p=1}^{P}}}\right)^{\#} \boldsymbol{y}. \end{aligned}  $$

If a set of *P*_pilot_, pilot symbols are available at subcarriers indexed by $\mathcal {P}=\{ p_{1},\dots,~p_{P_{\text {pilot}}}\}$, the intended transmit signal at antenna *q* can be represented as the sum of two signals:

46 $$ \begin{aligned} s_{q}^{p}(n) &= \sum_{i=1}^{P_{\text{pilot}}} S_{q}(p_{i})e^{j2\pi p_{i} n/N},\\s_{q}^{d}(n) &= \sum_{k \notin \mathcal{P}} S_{q}(k)e^{j2\pi k n/N}, \end{aligned}  $$

where the first sequence $s_{q}^{p}(n)$ contains the pilot symbols and the second sequence $s_{q}^{d}(n)$ contains the unknown data symbols transmitted by other intended transmitter. Then, the received vector in (42) is rearranged as follows:

47 $$ \begin{aligned} \boldsymbol{y} &= \boldsymbol{\Psi}_{i} \left(\left(\boldsymbol{x}_{i} + \left(\boldsymbol{I}_{N} \otimes \boldsymbol{K}\right)\boldsymbol{x}_{i}^{*} + \sum_{p=1}^{P}\left(\boldsymbol{I}_{N} \otimes \boldsymbol{A}_{p}\right) \boldsymbol{x}_{ip,p} \right) \otimes \boldsymbol{I}_{4N_{t}N_{r}} \right)\\ &\quad\boldsymbol{c} + \boldsymbol{\Psi}_{s} \left(\left(\boldsymbol{s}^{p} + \boldsymbol{s}^{d}\right) \otimes \boldsymbol{I}_{4N_{t}N_{r}} \right)\boldsymbol{c} + \boldsymbol{w}. \end{aligned}  $$

where ***s*** ^*p*^ and ***s*** ^*d*^ are constructed in the same way as ***s*** and contain the pilot symbols and unknown symbols, respectively. The initial estimate of ***c*** is modified to incorporate the pilot symbols as

48 $$ \widehat{\boldsymbol{c}}_{0} = \Big(\boldsymbol{\Psi}_{i} (\boldsymbol{x}_{i} \otimes \boldsymbol{I}_{4N_{t}Nr}) + \boldsymbol{\Psi}_{s} (\boldsymbol{s}^{p} \otimes \boldsymbol{I}_{4N_{t}N_{r}}) \Big)^{\#} \boldsymbol{y},  $$

and the estimates of ***s*** ^*d*^, ***K*** and ***A***_*p*_ at iteration *k* are given by

49 $$ \begin{aligned} \left(\begin{array}{l} \widehat{\boldsymbol{s}}_{k}^{d} \\ \widehat{\boldsymbol{k}}_{k} \\ \widehat{\boldsymbol{\alpha}}_{1,k} \\ \vdots \\ \widehat{\boldsymbol{\alpha}}_{P,k} \! \end{array}\right) & =\Big[\boldsymbol{\Psi}_{s} \widehat{\boldsymbol{C}}_{k-1},~\boldsymbol{\Psi}_{i} \Big(\text{diag}(\boldsymbol{x}_{i}^{*})\boldsymbol{B} \Big)\\ &\quad\otimes \widehat{\boldsymbol{c}}_{k-1},~\boldsymbol{\Psi}_{i} \Big(\text{diag}(\boldsymbol{x}_{ip,1}) \boldsymbol{B} \Big) \! \otimes \! \widehat{\boldsymbol{c}}_{k-1},\dots, \\ & \quad\boldsymbol{\Psi}_{i} \Big(\text{diag}(\boldsymbol{x}_{ip,P}) \boldsymbol{B} \Big) \otimes \widehat{\boldsymbol{c}}_{k-1} \!\Big]^{\#} \\ &\quad\times\Big(\boldsymbol{y} - \boldsymbol{\Psi}_{i} \widehat{\boldsymbol{C}}_{k-1} \boldsymbol{x}_{i} - \Psi_{s} \widehat{\boldsymbol{C}}_{k-1} \boldsymbol{s}^{p} \Big). \end{aligned}  $$

As before, $\widehat {\boldsymbol {s}}_{k}^{d}$ is converted to the frequency domain, demodulated then transformed to the time domain to obtain $\widetilde {\boldsymbol {s}}_{k}^{d}$. The updated estimate of ***c*** at iteration *k* is obtained as:

50 $$ \begin{aligned} \widehat{\boldsymbol{c}}_{k} &= \left(\boldsymbol{\Psi}_{i} \left(\left(\boldsymbol{x}_{i} + \left(\boldsymbol{I}_{N} \otimes \widehat{\boldsymbol{K}}_{k}\right)\boldsymbol{x}_{i}^{*} + \sum_{p=1}^{P}\left(\boldsymbol{I}_{N} \otimes\widehat{\boldsymbol{A}}_{3,p}\right) \boldsymbol{x}_{ip,p} \right) \otimes \boldsymbol{I}_{4N_{t}N_{r}} \right)\right.\\ &\qquad\left.+ \boldsymbol{\Psi}_{s} \left(\left(\boldsymbol{s}^{p} + \widetilde{\boldsymbol{s}}_{k}^{d}\right) \otimes \boldsymbol{I}_{4N_{t}N_{r}}\right) {\vphantom{\sum_{p=1}^{P}}}\right)^{\#} \boldsymbol{y}. \end{aligned}  $$

In the following, we summarize the different steps of the proposed algorithm:

1. Compute the augmented covariance matrix $\boldsymbol {R}_{\widetilde y}$ by time averaging of *T* received samples as:
  $$ \widehat{\boldsymbol{R}}_{\widetilde y} = \frac{1}{T} \sum_{t=1}^{T} \left(\begin{array}{l} \boldsymbol{y}_{t} \\ \boldsymbol{y}^{*}_{t} \end{array}\right) \left(\begin{array}{l} \boldsymbol{y}_{t} \\ \boldsymbol{y}^{*}_{t} \end{array}\right)^{H}   $$
2. Perform eigendecomposition of $\boldsymbol {R}_{\widetilde y}$ and take the *p* eigenvectors ***ν***_*i*_ corresponding to the smallest eigenvalue of $\boldsymbol {R}_{\widetilde y}$.
3. Construct the matrix $\boldsymbol {\overline \Theta }$ from ***ν***_*i*_ and compute the 4*N*_*t*_ *N*_*r*_ singular vectors of $\boldsymbol {\overline \Theta }$ corresponding to the zero singular value to form $\overline {\boldsymbol {\Phi }}$.
4. Build the matrices $\boldsymbol {\check \Phi _{i}}$ and $\boldsymbol {\check \Phi _{s}}$ as given in (39).
5. Estimate the ambiguity vector ***c*** by iterating between (44) and (45) if no pilot symbols are available or between (49) and (50) if a set of pilot symbols are available from the intended transceiver.

## Simulation results

In this section, we provide some simulation results on the performance of the proposed estimation algorithm for a 2×2 MIMO full-duplex system. The transmitted bits are mapped to 4-QAM symbols, then passed through an OFDM modulator of length *N*=64. The wireless channel is represented as a Rayleigh multipath fading channel with five equal-variance resolvable paths. Since the exact number of paths is supposed to be unknown, the algorithm is parametrized as if there are eight paths. In the following, the SNR is defined as the average intended-signal-to-thermal noise power ratio and the estimation mean square error (MSE) of ***H*** is $\textrm {MSE} = E\Big (||\boldsymbol {H} - \widehat {\boldsymbol {H}}||^{2}\Big).$ To model the RF impairments, a complete transmission chain is simulated. The PA coefficients are derived from the intercept points by taking the IIP 3=20 dBm. For the IQ mixer, the ratio between the direct signal and the image is set to 28 dB which is specified in 3GPP LTE specifications [23]. The ADC is modelled as a 14-bit quantizer to incorporate the quantization noise. Therefore, no simplifications are made regarding the different impairments. Antenna separation can attenuate the SI by 40 dB while the RF cancellation stage reduces the direct path by 30 dB [1] leaving the weaker reflections and transceiver impairments to be reduced by the proposed digital algorithm.

**Fig. 2:**
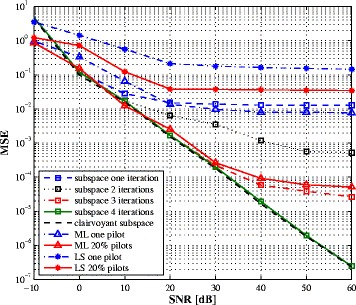
SI channel estimation MSE vs. SNR with 60 received OFDM symbols

**Fig. 3:**
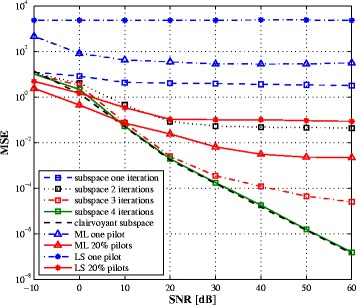
Intended channel estimation MSE vs. SNR with 60 received OFDM symbols

The proposed algorithm is compared to different channel estimators: the least square (LS) and the maximum likelihood (ML) algorithms. For the LS estimator, the channel coefficients are obtained using the known *self* signal and the pilot symbols in the intended signal. It simply considers the unknown symbols as additive noise. The ML estimate is obtained by maximizing the following cost function:

$${ \begin{aligned} L\left(\boldsymbol{H}^{(i)},~\boldsymbol{H}^{(s)}\right) &= \log \left(\det(\boldsymbol{R})\right)- \left(\boldsymbol{y} - \boldsymbol{H}^{(i)}\boldsymbol{x}-\boldsymbol{H}^{(s)}\boldsymbol{s}^{p}\right)^{H}\\ &\quad\times\boldsymbol{R}^{-1} \left(\boldsymbol{y} - \boldsymbol{H}^{(i)}\boldsymbol{x}-\boldsymbol{H}^{(s)}\boldsymbol{s}^{p}\right), \end{aligned}} $$

where $\boldsymbol {R} = \alpha ^{2} {\boldsymbol {H}^{(s)}}^{H}\boldsymbol {H}^{(s)} + \sigma ^{2} \boldsymbol {I}_{N_{r}M}$. An iterative procedure to find the ML estimate was proposed in [35]. The covariance matrix is obtained by averaging 60 OFDM blocks. Figures 2 and 3 plot the MSE vs. SNR curves for the SI and intended channel estimations, respectively. In both figures, one pilot symbol, from the intended transceiver, is used to solve the ambiguity matrix. For comparison purpose, a perfect estimate of the ambiguity term ***c*** is obtained as $\boldsymbol {c}_{perfect} = \arg \min _{\boldsymbol {c}}||\boldsymbol {\check h} - \boldsymbol {\Phi }\boldsymbol {c}||_{2}^{2}$ and the corresponding curves are labelled by clairvoyant subspace. It is seen that, when one pilot symbol is used in the ML and LS estimators, the proposed subspace algorithm offers notably lower MSE over a large SNR range. We also represent the performance of the ML and LS estimators when 20% of the transmit symbols are known (pilot symbols equally spaced within one OFDM symbol) while keeping one pilot symbol for the subspace method^4^. In this case, the three algorithms give comparable performance at low SNR region with the expanse of lower bandwidth efficiency. As the SNR increases, the performance of the LS and ML estimators saturate due to the reduced number of pilot symbols and the presence of the unknown transmit signal from the intended transceiver which acts as an additive noise. While the subspace algorithm exploits the information bearing in the unknown data to find the signal subspace. The ambiguity term is first solved using the known transmit symbols, then the iterative decoding ambiguity estimation is applied to improve the estimation performance. From Figs. 2 and 3, three to four iterations are sufficient to converge and the performance is close to the performance when the ambiguity term ***c*** is perfectly obtained. Note that the ML solution is also obtained in an iterative way and for a fair comparison; we simulate the performance of the ML estimator after four iterations. As it can be expected, the estimate of the SI channel is more accurate than the estimate of the intended channel. This can be explained by the fact that the self-signal is known while one pilot symbol is known in the intended signal.

**Fig. 4:**
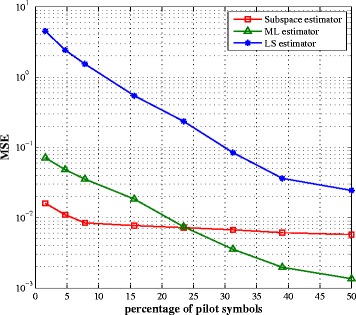
SI channel estimation MSE vs. percentage of pilot symbols for SNR = 10 dB

**Fig. 5:**
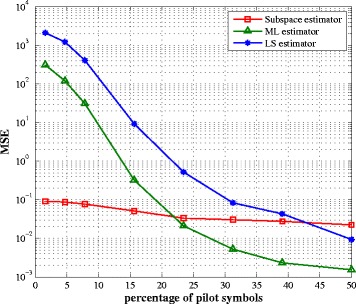
Intended channel estimation MSE vs. percentage of pilot symbols for SNR = 10 dB

The number of pilot symbols is a critical issue in channel estimation since a large pilot sequence provides better estimation performance but reduces the bandwidth efficiency of the system. In Figs. 4 and 5, we compare the impact of the number of pilot symbols on the performance of the three estimators. We periodically place the pilot symbols within an OFDM symbol. Optimal pilot placement requires to verify all *P*_pilot_ combinations from *N* subcarriers and hence, leads to an NP-hard problem beyond the scope of this paper, and is left for future work. It can be seen from these figures that the subspace method is not greatly affected by the number of pilot symbols since the subspaces are obtained using the second-order statistics of the received signal and not the transmit signal itself. Clearly, the proposed algorithm outperforms the ML and LS estimators at a reduced number of pilots while this tendency is inverted when the number of pilots increases. However, a system with a large amount of pilot symbols is not of practical interest.

**Fig. 6:**
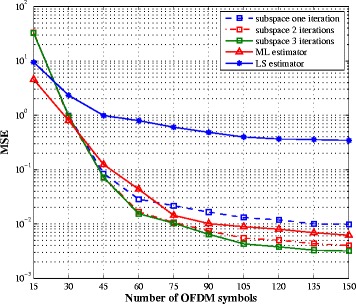
SI channel estimation MSE vs. number of OFDM symbols for SNR = 10 dB and one pilot symbol

**Fig. 7:**
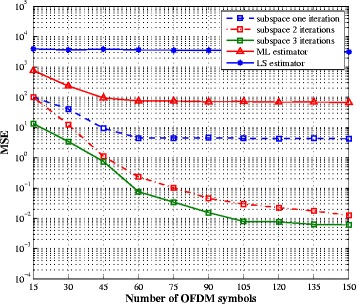
Intended channel estimation MSE vs. number of OFDM symbols for SNR = 10 dB and one pilot symbol

In Figs. 6 and 7, we evaluate the impact of the number of observed OFDM symbols on the estimation performance. For the three algorithms, we consider the transmission scheme where the number of pilot symbols is set to one and the SNR is 10 dB. As the subspace algorithm is based on estimates of the second-order statistic of the received signal, its performance varies with the number of OFDM symbols. All three algorithms are able to estimate the SI channel with an error floor for the LS. The ML and subspace algorithms offer the similar performance. On the other hand, the LS estimator fails to recover the intended channel, for any number of OFDM symbols. This can be explained by the fact that the number of unknowns (intended channel coefficients) is larger than the number of pilot symbols. Hence, it is not possible to use this method when the number of pilot symbols is small. The ML estimator presents also poor estimation performance for the intended channel, while the subspace method is able to return a good channel estimate, with a better bandwidth efficiency compared to the other estimators, as soon as there are enough OFDM symbols to compute the covariance matrix.

**Fig. 8:**
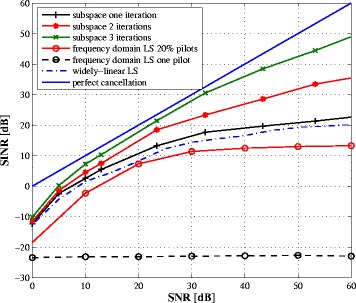
Output SINR vs. input SNR after SI cancellation

Our primary motivation of this work is to develop an accurate channel estimator to cancel the SI signal. The performance of the SI-canceller are represented by its achieved output signal-to-residual-SI-and-noise power ratio (SINR) after SI cancellation vs. the input SNR. Ideally, if SI could be completely cancelled then the residual SI after cancellation is 0, and consequently, the output SINR equals the input SNR as shown by the dashed line “perfect cancellation” in Fig. 8. In other words, the “perfect cancellation” is considered as the ideal upper-bound for the SINR. As shown in Fig. 8, with three iterations, the proposed subspace-based SI-canceller can offer an output SINR very close to the upper-bound over a large SNR range. At low SNR, the large estimation error results in a larger residual SI after cancellation, which ultimately affects the output SINR.

**Fig. 9:**
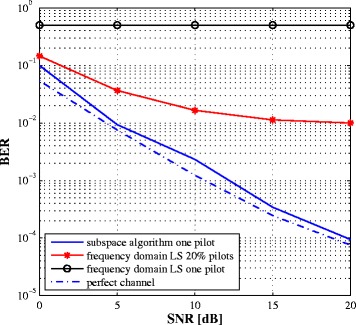
BER vs. SNR comparison of the proposed and the frequency domain LS techniques

We also investigate in Fig. 8 a frequency domain method to estimate the different parameter using the pilot symbols on some subcarriers. We resort to the LS estimator to find the channel responses at the pilot subcarriers. Since the remaining subcarriers contain unknown symbols from the intended transceiver, the complete channel responses are obtained by linear interpolation of the estimated coefficients. Thus, the frequency domain approach uses only the portion of the signal containing pilots while the proposed approach exploits the whole received signal through the second-order statistics. Clearly, the performance of the frequency domain approach highly depends on the number of pilots (as shown in Fig. 8) since the interpolation cannot model the variance of the channel in the frequency domain. We also compare the proposed method with the widely linear LS estimator in [26]. Note that the algorithm in [26] ignores the PA nonlinearities and does not incorporate the intended signal in the estimation process. Some time frames are dedicated to transmit orthogonal pilot symbols for estimation purpose, where the transceiver receives only its own signal. Therefore, the widely linear LS estimator incurs an overhead and requires synchronization between the two transceivers. Besides, it shows a noise floor at high SNR because the PA nonlinearity is not considered during the estimation process. On the other hand, by exploiting the whole received signal through its second-order statistics, the proposed method offers good performance even with one pilot and still outperforms the frequency domain approach (even with much larger number of pilots). Figure 9 plots the bit error rate (BER) vs. SNR curves of the two approaches. For comparison, we include the case of perfect channel estimate. To improve the BER, the SINR should be kept as high as possible at the demodulator. To conclude, while the frequency domain approach is more intuitive, it needs a large number of pilots and is outperformed by the proposed method.

**Fig. 10:**
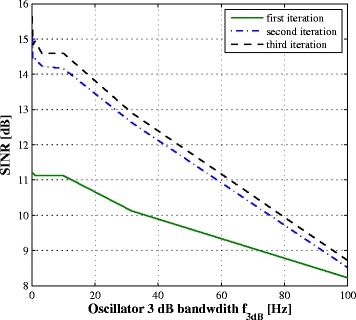
SINR after SI cancellation vs. *f*_3*dB*_

**Fig. 11:**
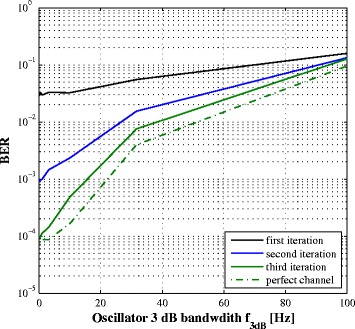
BER vs. phase noise *f*_3*dB*_

We evaluate the performance of the system in the presence of phase noise by simulation. Figures 10 and 11 plot respectively the SINR and the BER vs. the phase noise 3 dB bandwidth *f*_3*dB*_ for SNR =20 dB and common oscillator at the transmitter and the receiver. The residual SI depends obviously on the quality of the oscillator represented by its *f*_3*dB*_. Higher *f*_3*dB*_ results in a fast varying process. Clearly, the proposed method still offers good cancellation performance, which is degraded as *f*_3*dB*_ increases.

**Fig. 12:**
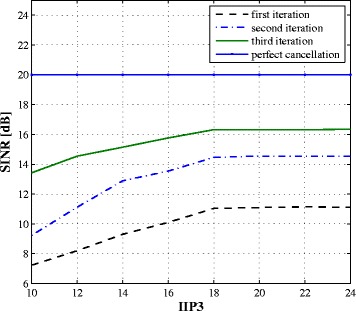
SINR after SI cancellation vs. PA IIP3

The PA nonlinearity effects on the performance of the proposed algorithm are also investigated through simulations. Figure 12 plots the resulting SINR after cancellation vs. the value of the PA third-order intercept point (IIP3) for SNR =20 dB. For *perfect* cancellation, the resulting SINR after cancellation would be the SNR =20 dB. A lower IIP3 indicates higher PA distortions (or poorer PA) and hence reduces the resulting SINR after cancellation. Figure 12 shows that as the IIP3 value increases, the cancellation performance is improved. However, for a sufficiently high IIP3 (e.g., 18 dBm or higher), the PA distortions are no longer dominant and the resulting SINR after cancellation is unchanged. This can be explained by the fact that, when developing the algorithm, the third-order component of the signal $x_{q,ip3}(n) = x_{q}^{IQ}(n)|x_{q}^{IQ}(n)|^{2}$ is approximated by *x*_*q*_(*n*)|*x*_*q*_(*n*)|^2^ to simplify the algorithm. This approximation only affects the algorithm performance when the nonlinear coefficients are sufficiently high.

## Conclusions

In this paper, a subspace-based estimation has been proposed to jointly estimate the SI channel, the intended channel and the transmitter impairments for MIMO full-duplex systems. By exploiting the covariance and pseudo-covariance matrix of the received signal, an effective way has been formulated to apply the subspace method for symmetric MIMO systems. The complete characterization of the second-order statistic of the received signal avoids the need of oversampling, required in traditional subspace methods. The subspace that contains the channels is blindly estimated and a short pilot sequence is needed to extract the channel coefficients from this subspace. The proposed method dramatically reduces the number of pilot symbols needed to identify the channel coefficients. Simulation results show that one pilot symbol is enough to obtain an accurate estimate while other methods are not able to recover the channel.

## Endnotes

^1^ The length of the cyclic prefix *N*_*cp*_ should be larger than the delay spread of the channel to eliminate the inter-symbol interference and inter-carrier interference. Therefore, if we know the length of the channel, we can set the cyclic prefix to be sufficiently large to satisfy *N*_*cp*_>*L*. Since this information is in general not available, *N*_*cp*_ is chosen to guarantee *N*_*cp*_>*L*. For example, if the distance between the two transceivers is 1 km, a cyclic prefix of 4 microsec is sufficient.

^2^ Physically, the additive noise arises from the thermal agitation of the charge carriers in an electronic device and is independent from the input. It can also contain interference from other systems whose signals are independent from the transmit signal of the considered system.

^3^ The previous condition is verified for independent channels between different antennas.

^4^ The pilot symbols are equally spaced within one OFDM symbol.

## Appendix 1: Eigenvalues of $\boldsymbol {R}_{\widetilde u}$

Following the discussion in Section 3, we mention that ***M*** is of rank *N*, then it has *N* strictly positive eigenvalues, *τ*_1_, *τ*_2_,…, *τ*_*N*_, and eigenvalue 0 of multiplicity *N*. And since the covariance matrix $\boldsymbol {R}_{\widetilde u}$ is given by $\alpha ^{2} \boldsymbol {M} \otimes \boldsymbol {I}_{2N_{t}}$, it follows that $\boldsymbol {R}_{\widetilde u}$ has also *N* eigenvalues *τ*_1_, *τ*_2_,…, *τ*_*N*_ each of multiplicity 2*N*_*t*_ and eigenvalue 0 of multiplicity 2*NN*_*t*_. To find the non-zero eigenvalues, we solve the characteristic polynomial of ***M*** given by

51 $$\begin{array}{@{}rcl@{}} \det\Big(\boldsymbol{M} - \tau \boldsymbol{I}_{2N}\Big) = 0. \end{array} $$

First, if *τ*=1 is an eigenvalue of ***M***, then it exists a vector ***a***≠**0** such that ***M*** ***a***−***a***=**0**. It follows that ***a***(1)=***a***(2)=⋯=***a***(2*N*)=0, which is in contradiction with ***a***≠**0**. Therefore, 1 is not an eigenvalue of ***M***.

By writing ***M*** as a block matrix:

52 $$ \boldsymbol{M} = \left(\begin{array}{ll} \boldsymbol{I}_{N} & \boldsymbol{M}_{1,2} \\ \boldsymbol{M}_{1,2} & \boldsymbol{I}_{N} \end{array}\right),  $$

the characteristic polynomial of ***M***, for *τ*≠1, is written as

53 $$ \begin{aligned} \det\left(\boldsymbol{M} - \tau \boldsymbol{I}_{2N}\right) & =\det\left((1-\tau)\boldsymbol{I}_{N}\right)\\ &\quad\times\det\left((1-\tau)\boldsymbol{I}_{N} - \boldsymbol{M}_{1,2} (1-\tau)^{-1}\boldsymbol{I}_{N} \boldsymbol{M}_{1,2}\right) \\ & =(1-\tau)^{N} \Big(1-\tau - (1-\tau)^{-1}\Big)^{N}, \end{aligned}  $$

where we used the fact that ***M***_1,2_ ***M***_1,2_=***I***_*N*_. Then, the solutions to det(***M***−*τ* ***I***_2*N*_)=0 are 0 and 2. Therefore, all non-zero eigenvalues of ***M*** are equal to 2 and thus all the non-zero eigenvalues of $\boldsymbol {R}_{\widetilde u}$ are equal to 2*α* ^2^.

## Appendix 2: Precoding for complex modulation

To make it simple, we consider the matrices ***P*** and ***Q*** having the following block structure:

$$\begin{array}{@{}rcl@{}} \boldsymbol{P} = \left(\begin{array}{ccccccccc} a \boldsymbol{I}_{N/2} & 0 \boldsymbol{I}_{N/2} \\ 0 \boldsymbol{I}_{N/2} & b \boldsymbol{I}_{N/2} \end{array} \right), \end{array} $$

54 $$\begin{array}{@{}rcl@{}} \boldsymbol{Q} = \left(\begin{array}{ccccccccc} 0 \boldsymbol{I}_{N/2} & c \boldsymbol{I}_{N/2} \\ d \boldsymbol{I}_{N/2} & 0 \boldsymbol{I}_{N/2} \end{array} \right), \end{array} $$

for given real numbers *a*, *b*, *c* and *d*. Similarly to the real modulation, we have $\boldsymbol {R}_{\widetilde u} = \boldsymbol {M} \otimes \boldsymbol {I}_{2N_{t}}$ where ***M*** for complex modulation is given by

$$\begin{aligned} \boldsymbol{M} & = \left(\begin{array}{cccc} \boldsymbol{P}\boldsymbol{P}^{T} + \boldsymbol{Q} \boldsymbol{Q}^{T} & \boldsymbol{P} \boldsymbol{Q}^{T} + \boldsymbol{Q} \boldsymbol{P}^{T} \\ \boldsymbol{P} \boldsymbol{Q}^{T} + \boldsymbol{Q} \boldsymbol{P}^{T} & \boldsymbol{P}\boldsymbol{P}^{T} + \boldsymbol{Q} \boldsymbol{Q}^{T} \end{array} \right) \\ & =\left(\begin{array}{cccc} (a^{2}+c^{2}) & 0 & 0 & (ad+bc) \\ 0 & (b^{2}+d^{2})& (ad+bc) & 0 \\ 0 & (ad+bc) & (a^{2}+c^{2}) & 0 \\ (ad+bc) & 0 & 0 & (b^{2}+d^{2}) \end{array} \!\right) \! \otimes \! \boldsymbol{I}_{N/2}, \end{aligned} $$

for *a* ^2^+*c* ^2^=*b* ^2^+*d* ^2^. Thus, for *a*, *b*, *c* and *d* satisfying *a* ^2^+*c* ^2^=*ad*+*bc* and *b* ^2^+*d* ^2^=*ad*+*bc*, each line of ***M*** is repeated two times and $\boldsymbol {R}_{\widetilde u}$ has rank 2*NN*_*t*_. As an example, we can take *a*=0.757, *b*=0.5032, *c*=0.4935 and *d*=0.7506.
